# Differential expression analysis for paired RNA-seq data

**DOI:** 10.1186/1471-2105-14-110

**Published:** 2013-03-27

**Authors:** Lisa M Chung, John P Ferguson, Wei Zheng, Feng Qian, Vincent Bruno, Ruth R Montgomery, Hongyu Zhao

**Affiliations:** 1 Department of Biostatistics, Yale School of Public Health, New Haven, Connecticut, USA; 2 Department of Statistics, George Washington University, Washington, DC, USA; 3 Novartis Institutes for BioMedical Research, Cambridge, Massachusetts, USA; 4 Section of Rheumatology, Yale School of Medicine, New Haven, Connecticut, USA; 5 Department of Microbiology and Immunology, University of Maryland School of Medicine, Baltimore, Maryland, USA

## Abstract

**Background:**

RNA-Seq technology measures the transcript abundance by generating sequence reads and counting their frequencies across different biological conditions. To identify differentially expressed genes between two conditions, it is important to consider the experimental design as well as the distributional property of the data. In many RNA-Seq studies, the expression data are obtained as multiple pairs, *e.g.*, pre- vs. post-treatment samples from the same individual. We seek to incorporate paired structure into analysis.

**Results:**

We present a Bayesian hierarchical mixture model for RNA-Seq data to separately account for the variability within and between individuals from a paired data structure. The method assumes a Poisson distribution for the data mixed with a gamma distribution to account variability between pairs. The effect of differential expression is modeled by two-component mixture model. The performance of this approach is examined by simulated and real data.

**Conclusions:**

In this setting, our proposed model provides higher sensitivity than existing methods to detect differential expression. Application to real RNA-Seq data demonstrates the usefulness of this method for detecting expression alteration for genes with low average expression levels or shorter transcript length.

## Background

Gene expression profiles are routinely collected to identify differentially expressed genes and pathways across various individuals and cellular states. Sequencing-based technologies offer more accurate quantification of expression levels compared to other technologies. Early sequence-based expression measured transcript abundance by counting short segments, known as tags, generated from the 3’ end of a transcript. Tag-based methods include the Serial Analysis of Gene Expression (SAGE, [1]), Cap Analysis of Gene Expression (CAGE), LongSAGE, and massively parallel signature sequencing (MPSS). The development of deep sequencing technology enables simultaneous sequencing of millions of molecules and has led to advanced approaches for expression measurement [2,3]. Digital gene expression - tag profiling [4] adapted the tag-based approach for use with the ‘next-generation’ sequencing platform. RNA-Seq is an alternative approach, that is an application of ‘whole genome shotgun sequencing’. Briefly, it entails generating a cDNA library by random priming off of fragmented RNA. The cDNA library is then subject to next-generation sequencing to generate short nucleotide sequences (reads) that correspond to the ends of the cDNA fragments. RNA-Seq aims to measure the entire transcriptome and is preferable to microarrays and tag-based approaches since it provides more information such as alternative splicing and isoform-specific gene expression with very low background signal and a wider dynamic range of quantification [5]. Moreover, recent experiments revealed that the RNA-Seq measures expression level with high accuracy and reproducibility [6-9].

Sequence-based approaches quantify gene expression as a ‘digital’ count and require modeling suitable for a count random variable. The Poisson distribution has been central in modelling expression data [10-12] and commonly applied to RNA-Seq data [6,13]. In particular, Li et al. (2012) proposed a permutation-based approach to generate the null distribution [14]. However, Poisson-based approaches may not take all the variations between biological samples into account. The Beta-Binomial hierarchical model [15,16], overdispersed logistic [17], and overdispersed log-linear models [18] were proposed to capture extra variance for each gene separately. Negative Bionomial models have been proposed to estimate the overdispersion parameter by shrinkage estimation [19-21], mean-dependent local regression [22], or empirically derived prior distribution [23]. Alternatively, beta-binomial [24] and Poisson mixture [25] models were proposed under the Bayesian modeling framework. Nonparametric method with resampling was also considered [26]. These approaches generally assume that samples under two groups are obtained independently. Recently, some of these approaches have been extended to deal with multi-factor design structures [14,16,21,22].

Many practical RNA sequencing studies collect data with a paired structure, where the global expression profiles are measured before and after a treatment is applied to the same individual. Appropriate modeling of such data requires taking this design structure as well as the distributional property of the data into account. The Poisson model has been used to test the effect of drugs when the observation occurs as paired data, such as predrug and postdrug counts [27]. Lee [28] considered a mixture model to account for extra variance among individuals over the level that would be expected under the Poisson model. These approaches assume independence of the paired observation conditional on the individual mean. Bivariate Poisson or negative binomial distribution are alternative choices to model correlations between observations [29,30].

In this paper, we propose a Bayesian hierarchical approach to modeling paired count data that separately accounts for the within and between individual variability from a paired data structure. Our work adopts the Poisson-Gamma mixture model [28] and utilizes a Bayesian approach to evaluate the expression difference. We note that the Bayesian models are widely utilized in microarray studies and have improved sensitivity to detect differential gene expression by sharing information among genes [31]. Mixture models are also commonly used to model differential expression, where non-differentially expressed and differently expressed genes correspond to different mixture components. Various mixture model specifications have been considered in the literature. The gamma and log-normal distribution were used to model the expression levels [32,33]. Smyth [34] assumed a point mass at zero for log scaled fold change for null genes and a normal distribution centered at zero for non-null genes. Lonnstedt et al. [35] and Gottardo et al. [36] proposed a mixture of two (null and non-null) or three normal (null, over, and under expression) distributions. Non-parametric approaches have also been utilized [31,37]. Lewin et al. [38] discussed various choices of mixture component priors and model checking.

The rest of this manuscript is organized as follows. Data Section introduces the biological problem and data that motivated this study. Methods Section presents our parametric model and the Bayesian method to identify genes with differential expression levels. The performance of the proposed model is examined by Simulations. Two sets of simulation studies are conducted: (1) those based on the model assumption to investigate the accuracy of the proposed method on parameter estimation, and (2) those based on mimicking the motivating data set to examine the robustness of the proposed method. Finally, the proposed method is applied to real data with detailed discussion of the results and comparisons with other methods.

## Data

Qian et al. (Qian F. et al.: Identification of genes critical for resistance to infection by West Nile virus using RNA-Seq analysis, submitted) designed an RNA-Seq experiment to study human West Nile virus (WNV) infection. One objective of this study was to identify altered genes/transcripts from viral infection of primary human macrophages in comparison to uninfected samples. This study naturally has a paired design structure. A total of 10 healthy donors were recruited according to the guidelines of the human research protection program of Yale University and cells were isolated from fresh heparinized blood samples for infection with WNV (strain CT 2741, MOI=1, for 24 hours) as described previously [39]. PolyA+ RNA was prepared from uninfected and WNV-infected primary macrophages, fragmented, and subjected to sequencing using the Illumina Genome Analyzer 2. Approximately 50 million quality filtered reads were obtained from each sample, and about 85% were mapped to the human transcriptome (hg19) with ENSEMBL transcript annotations (Release 57) using TOPHAT v.1.1.4 [40]. Genes and transcript isoforms were scored for expression by a maximum likelihood based method implemented in Cufflinks v.0.9.3 [41]. To analyze differential expression, the data were first converted from the FPKM unit (fragments per kilobase of exon per million fragments mapped) to the number of reads originated from each transcript isoform. The trimmed-mean method [42] was applied to further normalize the count expression values. The processed data contains transcript-level expression counts from a total of 20 samples consisting of 10 pairs of uninfected and virus infected samples. For differential expression analysis, we removed transcripts with less than 10 total counts across 10 uninfected samples or no observed count from 6 or more individuals in the uninfected conditions. After these steps, 37,111 transcripts were considered for data analysis.

## Methods

### Bayesian mixture model for paired counts

We now describe our Bayesian hierarchical mixture model to identify differentially expressed genes/transcripts from paired RNA-seq data. As noted above, such data arise naturally from experiments measuring the biological change from treatments. We start with an overdispersed count model [28]. The observations are denoted by a pair (*Y*_*gi*1_,*Y*_*gi*2_), for gene *g* = 1, …, *G* and individual *i* = 1, …, *n*, where *Y*_*gi*1 _is the observed baseline expression level and *Y*_*gi*2 _is the observed level after treatment. The sizes of the libraries are denoted as *N*_*i*1 _and *N*_*i*2_, respectively. Let *λ*_*gi *_denote the true baseline expression relative to the library size. Then, *Y*_*gi*1 _can be modeled as a Poisson random variable with mean *λ*_*gi*_*N*_*i*1_. Let *χ*_*g *_denote the expression level fold change after treatment so the true expression level is *χ*_*g*_*λ*_*gi*_*N*_*i*2_, then *Y*_*gi*2 _can be modeled as a Poisson random variable with mean *χ*_*g*_*λ*_*gi*_*N*_*i*2_. Our goal is to test whether there is any treatment effect, *i.e.*, *χ*_*g*_ ≠ 1, where

(1) $$\begin{array}{lll} Y_{\text{gi}1}|\lambda_{\text{gi}},\chi_{g} & ∼\text{Poisson}(N_{i1}\lambda_{\text{gi}}),\; & \;\\ Y_{\text{gi}2}|\lambda_{\text{gi}},\chi_{g} & ∼\text{Poisson}(N_{i2}\lambda_{\text{gi}}\chi_{g}).\; \end{array}$$

It has been shown that the variability among technical replicates for RNA-Seq data can be captured by the Poisson distribution [6]. However, greater variance can be expected, if observations are collected from individuals with differences in the underlying biological system. One way to model the overdispersion among the Poisson counts is to mix it with a Gamma distribution [28]. In this model, we use a Gamma distribution to model the baseline expected expression, *λ*_*gi*_, across individuals with shape parameter *α*_*g *_and rate *β*_*g*_;

(2) $$f_{\lambda }(\lambda_{\text{gi}})=\frac{\beta_{g}^{\alpha_{g}}}{\Gamma (\alpha_{g})}\lambda_{\text{gi}}^{\alpha_{g}-1}e^{-\beta_{g}\lambda_{\text{gi}}}.$$

This model allows us to obtain a simpler form of the predictive density, *i.e.*, the *λ*_*gi*_’s can be integrated out (see Appendix).

Assuming independence between the baseline expression and treatment effect, we use a two-component mixture model to characterize the fold change distribution, where the expression change state of each gene is defined by a latent variable *z*_*g*_, with *z*_*g*_ = 0 corresponding to no change and *z*_*g*_ = 1 otherwise. We assume that *z*_*g *_has a probability of *Π*_0 _for equal expression, *i.e.*, *z*_*g*_ = 0, and a probability of *Π*_1_ = 1 - *Π*_0 _for differential expression. Given a state, 0 or 1, the log-scaled fold change is assumed to follow a normal distribution. Under equal expression, the log-fold change is assumed to arise from a normal distribution centered at zero and variance $\sigma_{0}^{2}$. For genes with differential expressions, if we assume their log-fold changes follow a normal distribution centered around zero, we implicitly assume that there is equal chance for a gene to be either over or under expressed. However, more genes were under-expressed after the viral infection for the data set described earlier, with 3.2*%* of transcripts showing increased expression by more than 4 fold after the infection whereas 4.3*%* showing reduction by more than 4 folds. To accommodate this asymmetry, we assume the log-fold change for non-null genes arises from a normal distribution with mean *μ*_1_, which may be different from 0, and variance $\sigma_{1}^{2}$.

$$\begin{array}{ll} \log(\chi_{g})|(z_{g}=0) & ∼\text{Normal}(0,\sigma_{0}^{2})\\ \log(\chi_{g})|(z_{g}=1) & ∼\text{Normal}(\mu_{1},\sigma_{1}^{2}) \end{array}$$

**Figure 1:**
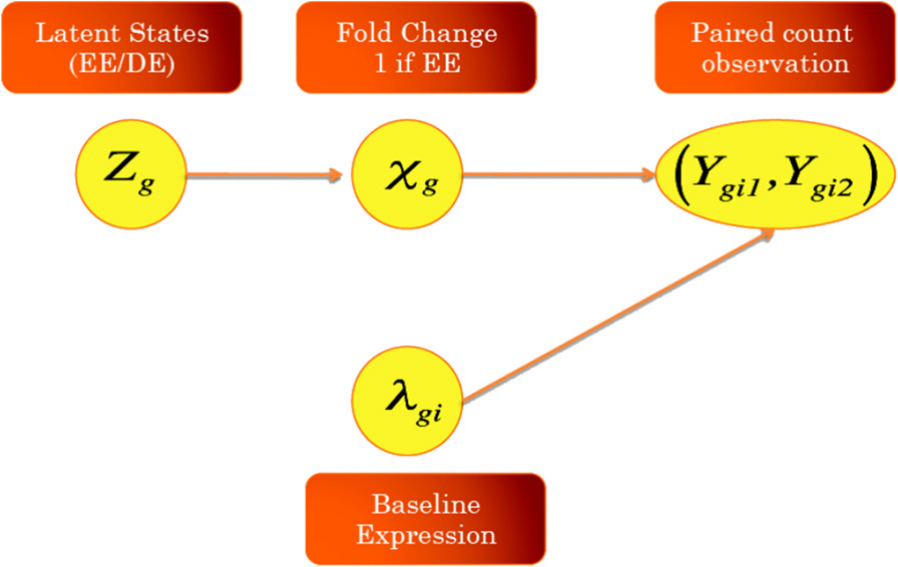
Diagram illustrating the hierarchical model for paired RNA-Seq data.

Collecting all the components discussed so far, the model can be summarized in Figure 1. Under this set-up, the goal is to estimate the posterior probability that a specific gene is differentially expressed after treatment, *i.e.*, *Pr*(*z*_*g*_ = 1|data). Genes can then be inferred to DE (Differential Expression) or EE (Equal Expression) according to these probabilities.

To complete our model description, we need to specify prior assumptions for the unknown model parameters, $\theta =(\{\alpha_{g}\},\{\beta_{g}\},\Pi_{0},\Pi_{1},\sigma_{0}^{2},\mu_{1},\sigma_{1}^{2})$. In our implementation, we assume non-informative priors for these unknown parameters: 

1. (*Π*_0_, *Π*_1_)∼ Dirichlet(1,1), *i.e.*, *Π*_0_ ∼ Uniform(0, 1).

2. Each *α*_*g *_and *β*_*g *_has a non-informative prior.

3. $p(\sigma_{0}^{2})\propto 1/\sigma_{0}^{2}$ and $p(\sigma_{1}^{2})\propto 1/\sigma_{1}^{2}$.

4. *μ*_1 _has an improper prior.

5. Joint independency among all the parameters.

### Parameter estimation via Markov chain Monte-Carlo (MCMC)

In this section, we describe the Gibbs sampling algorithm [43] that we use to iteratively sample model parameters from their conditional distributions given the other parameters and the observed data. First, we evaluate the conditional distribution of parameters (*α*_*g*_, *β*_*g*_) characterizing the baseline expression distribution (*λ*_*gi*_). These parameters are separately updated using the Metropolis-Hastings algorithm. For the latent state *z*_*g *_and expression level change *χ*_*g*_, the state *z*_*g *_is first proposed and then *χ*_*g *_is sampled given the state. Lewin et al. [38] discussed this type of move with various choices of the mixture distribution. Details of our updates on the pair of (*χ*_*g*_, *z*_*g*_) are described in the Appendix. Mixing proportions (*Π*_0_, *Π*_1_) and hyper-parameters for the mixture distribution ($\sigma_{0}^{2}$, $\sigma_{1}^{2}$, *μ*_1_) are sampled from their conditional posterior distributions which can be derived in closed forms.

### DE classification and false discovery rate estimation

The MCMC algorithm generates random samples from the joint posterior distribution of all model parameters. These samples are then used to infer the status of differential expression. One way to select a set of interesting genes is to rank genes using estimated posterior-mean fold change

(3) $$\hat{\chi_{g}}\approx \exp\left\{\frac{1}{T}\overset{T}{\underset{t=1}{\sum }}\log\left(\overset{\left(t\right)}{\underset{g}{\chi }}\right)\right\},$$

where *T* is the number of iterations used for inference after the burn-in period and $\chi_{g}^{\left(t\right)}$ is the sampled value for the fold change on iteration *t* of the Gibbs sampling algorithm. Another way to select DE genes is to consider the latent variable, *z*_*g*_. During the MCMC iteration, the expression state is sampled along with the fold change estimates. These MCMC samples can be used to approximate the posterior probability of differential expression by counting the proportion of sampled states being differentially expressed:

$$p_{g}=P(z_{g}=1|\text{data})\approx \frac{1}{T}\overset{T}{\underset{t=1}{\sum }}I\left(z_{g}^{\left(t\right)}=1\right).$$

The Bayes’ rule assigns a gene’s expression status according to the largest posterior probability. An alternative is to classify a gene if the posterior probability of being non-null is greater than a threshold (*p*_*thres*_) : *p*_*g*_ > *p*_*thres*_. For example, one choice would be *p*_*thres*_ = 0.5. The false discovery rate can be estimated from these posterior probabilities [31]:

(4) $$\hat{\text{FDR}}=\frac{1}{♯(p_{g}>p_{\text{thres}})}\underset{g:p_{g}>p_{\text{thres}}}{\sum }(1-p_{g})$$

The method was implemented in R and is available at http://bioinformatics.med.yale.edu.

## Results and discussion

### Simulations

#### Simulations based on the model assumptions

The first part of the simulation was conducted to examine the performance of the proposed approach when the data are generated under the model assumptions. For 10,000 genes and 10 individuals, we simulate expression counts both before and after treatment according to Equation 1. Library sizes are sampled uniformly from 7 to 18 millions and relative expected baseline expression *λ*_*gi *_are drawn from a Gamma distribution with shape 0.1 and rate 1,000. For simplicity, we consider a two-component log-normal mixture model for effect size. For the null genes (90%), the log-scaled effect is sampled from a normal distribution with a mean 0 and a standard deviation (*σ*_0_) 0.1, whereas the log-effects are sampled from a normal distribution with mean (*μ*_1_) of 1.5 and standard deviation (*σ*_1_) of 0.5 for the non-null genes. For the simulation studies, the true library sizes are used for the parameter estimation.

**Figure 2:**
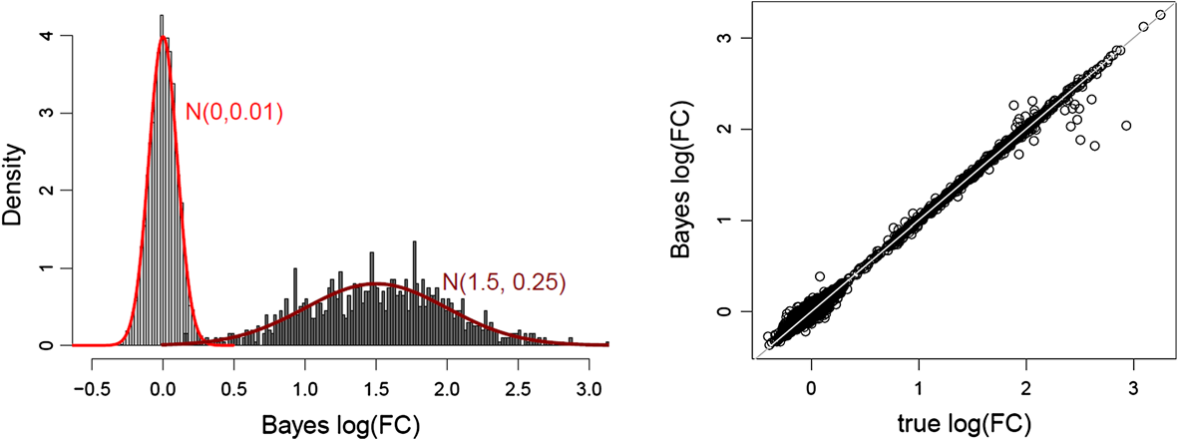
**Estimated fold change.** The left panel shows the distribution of the estimated fold changes under EE and DE by the Bayes’ rule. The red lines are the true fold change distributions. The right panel displays the relationship between the estimated and true fold change.

Results in Table 1 show that the proposed approach estimates the model paramters well. With a posterior probability cutoff of 0.5, the algorithm identified more than 97*%* of true DE genes with an FDR of approximately 1*%*. Figure 2 illustrates the estimated fold changes showing the good performance of our algorithm.

**Table 1:** Posterior means of the parameters in the model

**Parameters**	**True parameter**	**Posterior mean**
$\sigma_{0}^{2}$	0.01	0.013 (0.002)
*μ*_1_	1.5	1.501 (0.015)
$\sigma_{1}^{2}$	0.25	0.238 (0.015)
*Π*_1_	0.1	0.099 (0.001)

#### Simulations based on the empirical data

In the second part of the simulation, we assume that the expression abundance is measured for 5,000 genes simultaneously before and after a given treatment. The number of individuals is set to be 10 for the relatively larger sample case (cases 1 and 4), 5 for the medium (cases 2 and 5), and 3 for the relatively smaller sample case (cases 3 and 6). The size of each library is randomly sampled from 1.8 to 3 million to have simulated count distribution compatible with the real data distribution. The infected set of the RNA-seq data (Data Section, Qian F. et al. for details) was used as the expected baseline count data to mimic the observed mean-specific dispersion. First, we sample 5,000 gene indices with replacement to get the expected baseline expression. Expression counts from the selected indices are summarized by a matrix where rows from this data matrix correspond to the selected genes in the original data matrix and columns correspond to individuals. Then, the relative expression (*λ*_*gi*, *i* = 1, …, *N*_, Equation 1) is computed proportional to the total counts in each sample.

Among 5,000 genes, the first 4,000 are assumed to have no change (*z*_*g*_ = 0) and their log-fold changes, log(*χ*_*g*_), are sampled from a normal distribution with a mean of 0 and a variance of 4 × 10^-4^. For the rest of non-null genes, we considered the following two scenarios. An empirical set-up (cases 1, 2, 3) utilizes nominal fold change from the uninfected data set. Cases 4, 5, and 6 consider a theoretical setup, where the log-scaled fold change is drawn from a normal distribution with a mean of zero and a variance of 1. We further filter out non-null genes whose true fold changes are less than 1.4.

Each case was repeated 100 times. We compare the performance of our approach with DESeq (version 1.8.3) [22] and edgeR (version 2.6.10) [21], two widely used methods for RNA-seq data for the purpose of identifying differentially expressed genes. These two methods assume a negative binomial distribution to explain the variance due to the replicate. DESeq utilizes a smoothing curve to compute the overdispersion as a function of the average expression level. An option ‘pooled-CR’ is used to estimate the overdispersion parameter [44]. In edgeR, a common dispersion setting is used which assumes a consistent overdispersion across all the features and estimates the parameter using a common likelihood function. A paired design can be incorporated by utilizing generalized linear model. For each application, the true library sizes are used as the library size inputs.

**Figure 3:**
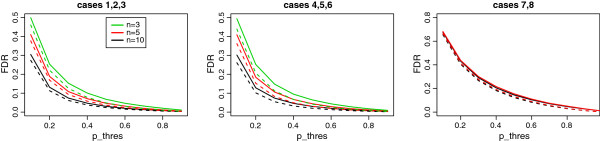
**False discovery rate from the simulation.** True and estimated false discovery rates are compared across different threshold for posterior probability. Solid lines are true values and dashed lines are estimated values averaged over all simulations. Left panel shows the result from simulation cases 1, 2, and 3, where non-null fold change is empirically generated. Results for cases 4, 5, 6 and 7,8 are illustrated on the middle panel and right panel, respectively.

**Figure 4:**
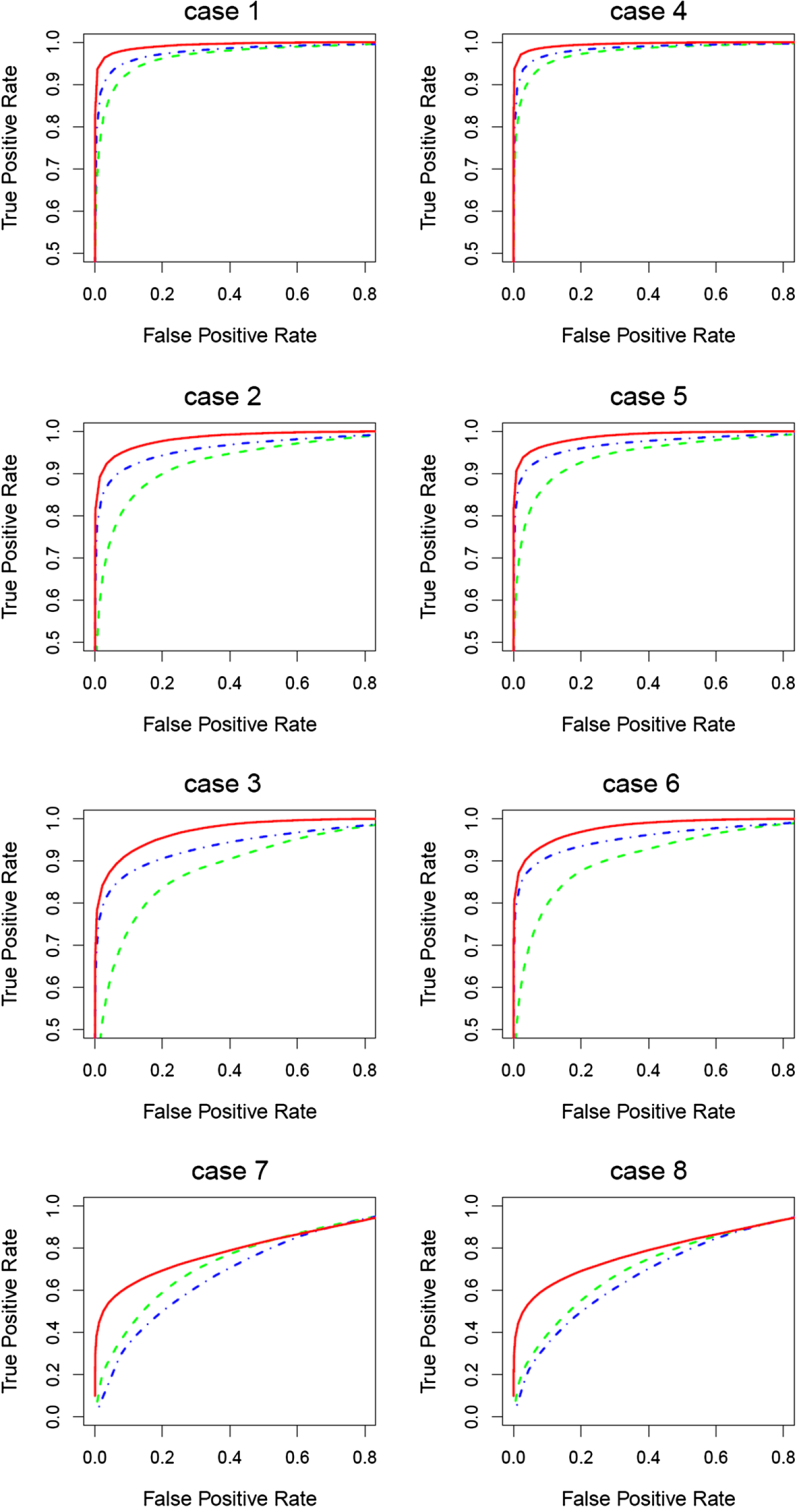
**Simulation results.** Operating characteristics for 8 simulation settings are plotted with red, green, and blue lines for the Bayes, DESeq, and edgeR methods, respectively.

Table 2 summarizes the results of our approach. Overall, we see excellent performance of our method in inferring the expression change status (reflected in a high correlation with the true status) as well as the parameters characterizing the distributions for the null and non-null genes. Since true expression states are known in the simulation, we call a feature to be differentially expressed if *p*_*g*_ > *p*_*thres *_and compare the estimated false discovery rate with the true value (Figure 3). The FDR is estimated well for cases with large sample sizes as *p*_*thres*_ increases, while it is slightly under-estimated for small sample sizes. Figure 4 illustrates the receiver operating characteristics averaged across 100 simulations under four different simulation settings. For each setting, the true positive rate is plotted against the false positive rate. The corresponding rates are computed by ranking genes from the largest posterior probability by the Bayesian approach (then, the largest fold change, if tied) or from the smallest p-value by each of the other methods. The Bayesian approach shows higher sensitivity at the same level of false positive rates than the edgeR and DESeq. Especially, the Bayesian model achieves better performance for smaller sample size and empirical fold change setting (case 2 or 3).

**Table 2:** Estimated posterior means and results for empirical simulation

	**Case 1**	**Case 2**	**Case 3**
***N***	**10**	**5**	**3**
*μ*_1_	-0.170 (0.037)	-0.169 (0.041)	-0.157 (0.041)
$\sigma_{0}^{2}$	3.653×10^-4^	3.604×10^-4^	3.83×10^-4^
	(3×10^-5^)	(4.421×10^-5^)	(6.090×10^-5^)
$\sigma_{1}^{2}$	0.984 (0.104)	0.968 (0.115)	0.955 (0.110)
*Π*_1_	0.151 (0.004)	0.153 (0.005)	0.156 (0.006)
$\text{cor}{\left(\chi_{g},\hat{\chi_{g}}\right)}^{*}$	0.972 (0.006)	0.993 (0.003)	0.953 (0.011)
*FDR*	0.030 (0.008)	0.046 (0.011)	0.068 (0.013)
$\hat{\text{FDR}}$	0.024 (0.004)	0.037 (0.005)	0.049 (0.006)
Sensitivity	0.928 (0.014)	0.866 (0.020)	0.802 (0.025)
Specificity	0.995 (0.001)	0.994 (0.002)	0.991 (0.002)
	**Case 4**	**Case 5**	**Case 6**
***N***	**10**	**5**	**3**
*μ*_1_	0.007 (0.035)	0.006 (0.038)	-0.002 (0.037)
$\sigma_{0}^{2}$	3.634×10^-4^	3.532×10^-4^	3.450×10^-4^
	(2.931×10^-5^)	(4.155×10^-5^)	(5.283×10^-5^)
$\sigma_{1}^{2}$	1.172 (0.048)	1.151 (0.059)	1.140 (0.050)
*Π*_1_	0.179 (0.003)	0.183 (0.004)	0.188 (0.005)
$\text{cor}{\left(\chi_{g},\hat{\chi_{g}}\right)}^{*}$	0.990 (0.002)	0.979 (0.004)	0.965 (0.007)
*FDR*	0.030 (0.008)	0.044 (0.009)	0.064 (0.012)
$\hat{\text{FDR}}$	0.021 (0.004)	0.031 (0.005)	0.042 (0.006)
Sensitivity	0.953 (0.011)	0.906 (0.015)	0.862 (0.020)
Specificity	0.995 (0.001)	0.992 (0.002)	0.989 (0.002)

Operating characteristics are based on the Bayes rule. $\text{cor}{\left(\chi_{g},\hat{\chi_{g}}\right)}^{*}$ is the correlation coefficient between the true difference and the estimated difference.

We further considered a simulation scenario similar with the real data. As shown in the data application, the log-scaled fold change estimated from the data has larger variance under null component. We set the null component variance to be 0.35 and repeated the simulation 50 times. For features in the non-null group, log-fold change was sampled from a normal distribution with a mean of -0.45 and a variance of 4. Simulation was performed with the sample size of 10 (case 7) and the size of 5 (case 8). Averages of the parameter estimates $\left(\mu_{1},\sigma_{0}^{2},\sigma_{1}^{2},\Pi_{1}\right)$ for cases 7 and 8 are (-0.42, 0.35, 3.92, 0.20) and (-0.42, 0.35, 3.85, 0.21), respectively. Similarly with the cases 1 through 6, the estimated false discovery rate is examined (Figure 3) and performance of the proposed approach is compared with two existing methods (Figure 4).

### Applications

#### Differential expression analysis with the Bayesian modeling

**Figure 5:**
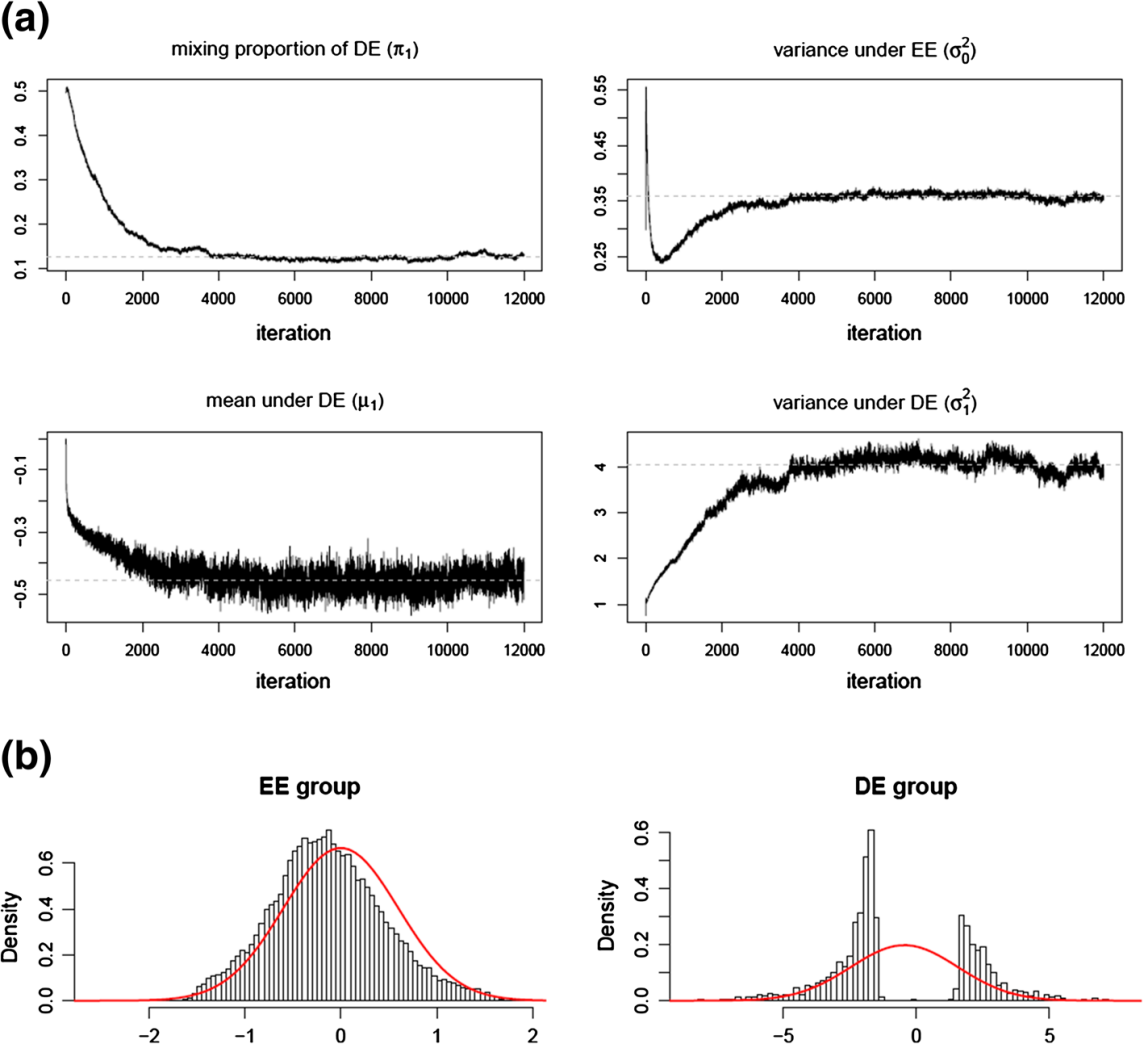
**Trace of parameters regarding the mixture distrubution.** Trace of parameters regarding the mixture distrubution **(a)** and distributions of fold change estimates for genes classified into EE and DE groups, respectively, by the Bayes’ rule **(b)**.

**Figure 6:**
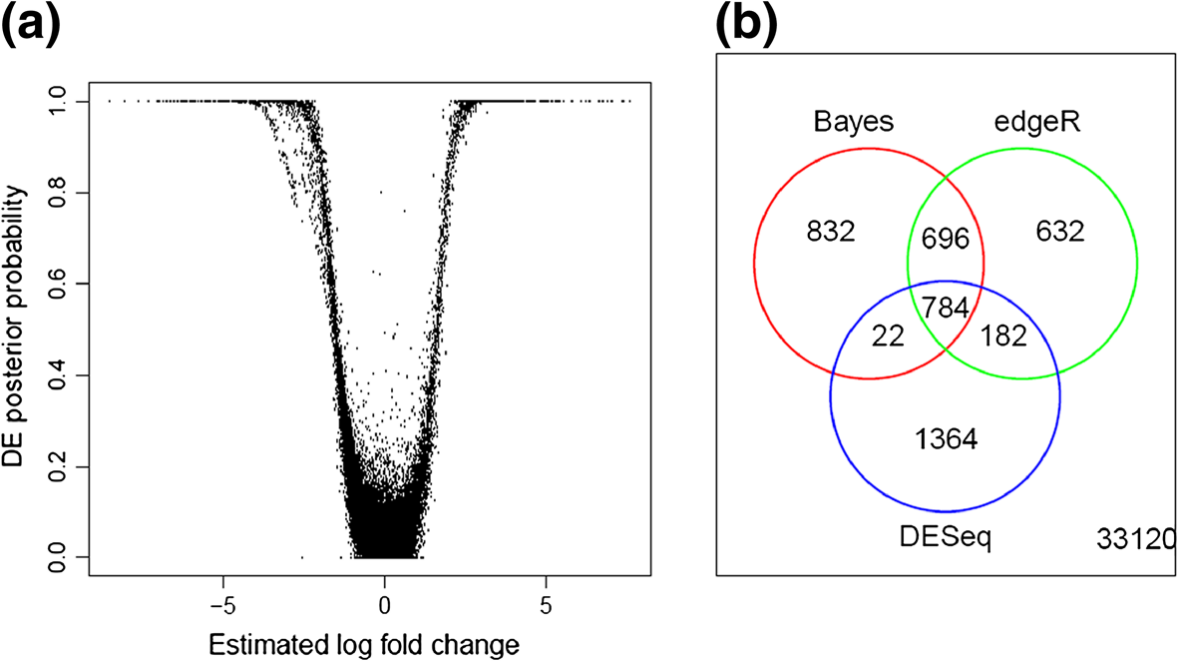
**Result of the Bayesian approach and comparison with other existing methods.** Posterior probabilities against estimated fold change **(a)** and consistency between the Bayesian approach and existing approaches when the same number of top-ranked transcripts are chosen **(b)**.

In this section, we apply our method to the motivating data set described in the Data Section. Initial values of the model parameters are calculated directly from the data. The MCMC sampling is run 4,000 iterations after discarding the first 8,000 iterations. On average, computational time was around 5 minutes per every 100 iterations. The number of total iterations and burn-in period are determined by monitoring trace plots of MCMC samples (Figure 5 (a)). We estimate the mixing proportion to be 0.88 and 0.12 for EE and DE group, respectively. The posterior means for the parameters *μ*_1_ and $\sigma_{1}^{2}$ are -0.45 and 4.04, respectively. The null group has a variance of 0.35. Under the Bayes rule (*p*_*thres*_ = 0.5), 2,352 transcripts are classified into DE after the West Nile virus infection. The estimated FDR is 16.2*%* from Equation 4. Figure 5 (b) illustrates the fold change distributions under DE and EE based on the Bayes rule classification. The estimated fold changes are plotted in Figure 6 (a) against their DE posterior probabilities.

#### Comparisons with existing methods

**Figure 7:**
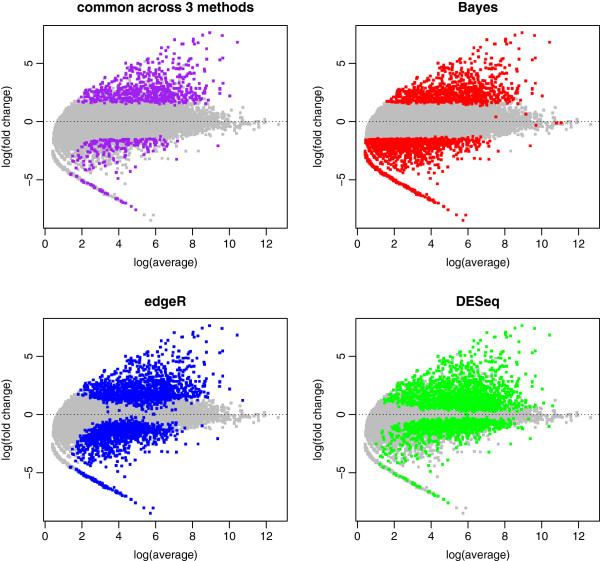
**Comparison of DE transcripts.** Commonly detected transcripts by all three methods are labeled in purple: log-scaled Bayesian estimated fold change against log-scaled average expression. Other three panels show DE transcripts detected by each of three methods. They are labeled in red, green, and blue for the Bayes, DESeq, and edgeR methods, respectively.

**Figure 8:**
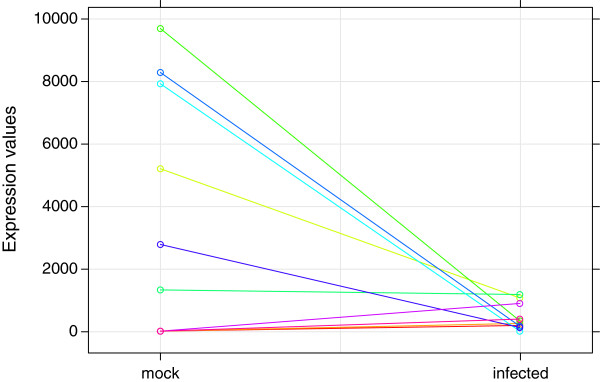
**Example of uniquely selected by the proposed Bayesian model.** Illustration of expresseion values from a transcript detected by the proposed method only.

**Figure 9:**
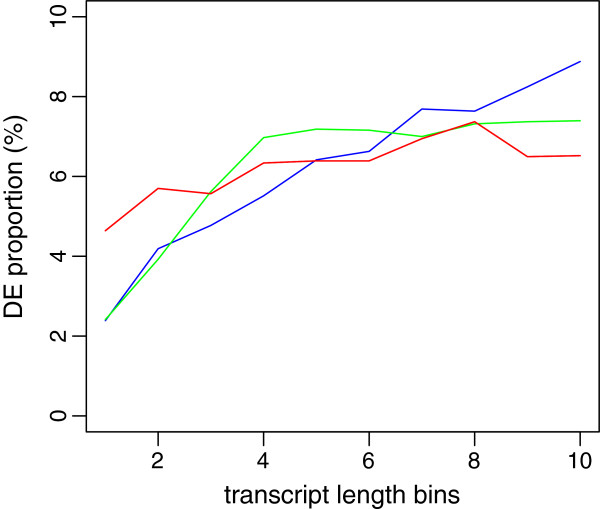
**DE proportion and transcript length.** Proportion of DE transcripts over their average expression level. Transcripts are partitioned into 10 equal-sized bins by their expression levels. The proportion of transcripts inferred to be DE is plotted on the y-axis. Red, green, and blue lines are from Bayes, DESeq, and edgeR methods, respectively.

In this section, we compare DE analysis results between our approach and existing methods. The DESeq or edgeR is applied to the same data set and top 2,352 DE transcripts are selected by their p-values. The edgeR shows higher consistency with our Bayesian model with 63.5% of overlap than the DESeq having 34.3% of overlapping transcripts. Specifically, 832, 632, and 1,364 transcripts are detected uniquely by the Bayes, edgeR, and DESeq, respectively (Figure 6). Our approach detects those having low average expression and high fold change. In contrast, other approaches tend to identify more transcripts with high expression level and low fold change (Figure 7). Transcripts which have evidence of differential expression only by the proposed model often have large inter-individual variation. Their fold changes are high after the treatment except a few low expressed individuals. Figure 8 illustrates an example of uniquely identified transcript by our proposed approach. This transcript is a product of SLAMF7, which is known to play a role in natural killer cell activation [45]. Another interesting feature of the proposed method is that the proportion of DE genes is consistent across transcript length. Among the bottom 10*%* of the short transcripts, 4.6*%* are detected by the proposed approach while 2.4*%* are found by other methods. Among the top 10*%* of the long transcripts, 6.5*%* are detected by the proposed method whereas 7.4 and 8.9*%* are detected by DESeq and edgeR, respectively. To investigate more details, Figure 9 illustrates the DE proportion when the transcripts partitioned into 10 equal-sized bins based on their length.

#### Bioinformatics annotations of the results

Pathway-level analysis is one effective way to summarize biological relevance of differentially expressed genes. We perform gene enrichment analysis using DAVID (http://david.abcc.ncifcrf.gov/). 2,352 DE transcripts inferred from our approach are mapped to 1,518 DAVID IDs for functional annotation clustering. Cluster 1 (DAVID enrichment score: 11.39) represents cellular response to the WNV infection. Specifically, pathways in cluster 10 (score: 2.72) are related to the activation of the macrophage after the virus infection. Molecular functions of these transcripts are characterized by their cytokine production (GO:0001817) in cluster 3. Cluster 8 (score: 2.89) consists of transcripts that are involved in apoptosis (GO:0042981) and the regulation of programmed cell death (GO:0043067). The induction of apoptosis by WNV is essential in the regulation of pro-inflammatory responses, and has been previously reported in cell lines and neuronal cell types [46,47]. These clusters and related top pathways are reported on Table 3 with enrichment scores and p-values.

**Table 3:** Selected pathways from the functional analysis

**Term**		**Count**	**p-value**
Cluster 1	score: 11.39		
Defense response	GO:0006952	106	5.3E-14
Response to wonding	GO:0006954	90	1.3E-11
Inflammatory response	GO:0009611	63	1.0E-10
Cluster 2	score: 5.43		
Response to molecule of lipopolysaccharide	GO:0002237	23	9.4E-8
Response to cytokine stimulus	GO:0034097	18	8.0E-5
Response to bacterium	GO:0009617	31	3.5E-4
Cluster 3	score: 5.19		
Regulation of cytokine production	GO:0001817	41	2.9E-9
Positive regulation of cytokine production	GO:0001819	20	8.0E-5
positive regulation of multicellular organismal process	GO:0051240	35	1.1E-3
Cluster 8	score: 2.89		
Regulation of apoptosis	GO:0042981	100	1.0E-5
Regulation of programmed cell death	GO:0043067	100	1.5E-5
Regulation of cell death	GO:0010941	100	1.8E-5
Cluster 10	score: 2.72		
Leukocyte activation	GO:0045321	41	9.2E-6
Cell activation	GO:0001775	46	1.1E-5
T cell activation	GO:0046649	26	2.0E-5

Count column indicates the number of DAVID IDs associated with each pathway.

## Conclusions

In this paper, we have presented a hierarchical mixture model for the identification of differential gene expression from RNA-Seq data motivated by a West Nile Virus study, which collected samples as multiple pairs, *i.e.* pre- vs. post-treatment for each individual. While such design is common in biological investigations, few existing methods analyze such data appropriately. With a hierarchical Bayesian mixture model coupled with inference through MCMC, our approach incorporates variability across genes, individuals, and treatment effects in the context of a paired experiment. Application to both simulated and real data demonstrates that our model and implementation is suitable for paired design, having distinct advantages compared to the existing methods.

Simulation study suggests that our Bayesian setting can have better power to detect differential gene expression. In the real data application, our proposed is able to identify transcripts with large treatment effects but low expression levels, whereas these transcripts were not inferred to be differentially expressed by other approaches. This is likely due to the more flexible and adaptable modeling of variance across individuals in our approach. Further examination of the characteristics of these top-ranked transcripts shows that the proportion of top-ranked transcripts in the short transcript group is consistent with the proportion in the long transcript group. On the other hand, the gene sets detected by the existing approaches show a bias towards longer transcripts, as has been noted in the literature before [48,49]. Our model reduces this bias and as a result facilitates detection of some short-length differentially expressed transcripts that the other approaches miss.

We have assumed that the log-fold change arises from a mixture of two normal distributions. Under DE, the model allows the mean of log-fold change distribution not to be restricted at zero. By doing so, our proposed model can be applied to the data showing asymmetry between over and under expression. A normal distributional assumption is shown to be robust from simulation study under empirical fold change scenarios. Other possible choices for the null genes include a point mass at 0 [50], uniform distribution around 0, and a log-Gamma distribution with a mean 0. Similar distributional assumptions can be made for the non-null genes under the two-component mixture set-up. Alternatively, one can consider a mixture of three components consisting of equal, over, and under expression states. Further extension can be considered by allowing variation in the magnitude of expression change across individuals.

## Appendix

### Variability across individuals

The Poisson-Gamma setting (Equation 1 and 2) allows extra variance among count expression values [28]. The variance of the count is given as

$$\begin{array}{lll} \text{Var}(Y_{\text{gi}1}) & =E(\text{Var}(Y_{\text{gi}1}|\lambda_{\text{gi}}))+\text{Var}(E(Y_{\text{gi}1}|\lambda_{\text{gi}}))\; & \;\\ & =E(N_{i1}\lambda_{\text{gi}})+\text{Var}(N_{i1}\lambda_{\text{gi}})\; & \;\\ & =\frac{N_{i1}\alpha_{g}}{\beta_{g}}\left(1+\frac{N_{i1}}{\beta_{g}}\right).\; & \; \end{array}$$

### Modeling details

The joint density of *z*_*g*_ and *χ*_*g*_ is

$$\begin{array}{lll} p(\chi_{g},z_{g}) & ∼\Pi_{0}\text{LogNormal}(0,\sigma_{0}^{2})I(z_{g}=0)\; & \;\\ & \;+\Pi_{1}\text{LogNormal}(\mu_{1},\sigma_{1}^{2})I(z_{g}=1).\; & \; \end{array}$$

Let *θ* be a vector of all model parameters, $\theta =\left(\left\{\alpha_{g}\right\},,,\left\{\beta_{g}\right\},,,\pi_{0},,,\pi_{1},,,\sigma_{0}^{2},,,\mu_{1},,,\sigma_{1}^{2}\right)$. The complete likelihood of the model is

$$\;\begin{array}{l} p(Y,\chi ,z|\theta )=\underset{g}{\prod }p(Y_{g},\chi_{g},z_{g}|\theta )\\ \;=\underset{g}{\prod }p(Y_{g}|\chi_{g},z_{g},\theta )p(\chi_{g}|z_{g},\theta )p(z_{g}|\theta )\\ \;=\underset{g}{\prod }\left\{\underset{i}{\prod }p(Y_{\text{gi}}|\chi_{g},z_{g},\theta )\right\}p(\chi_{g}|z_{g},\theta )p(z_{g}|\theta )\\ \;=\underset{g}{\prod }\left\{\underset{i}{\prod }\int p(Y_{\text{gi}}|\lambda_{\text{gi}},\chi_{g},z_{g},\theta )p(\lambda_{\text{gi}}|\theta )d\lambda_{\text{gi}}\right\}\\ \;\times p(\chi_{g}|z_{g},\theta )p(z_{g}|\theta ) \end{array}$$

Here, some details on the integral over *λ*_*gi*_ follow.

$$\begin{array}{lll} & \int p(Y_{\text{gi}}|\lambda_{\text{gi}},\chi_{g},z_{g},\theta )p(\lambda_{\text{gi}}|\theta )d\lambda_{\text{gi}}\; & \;\\ = & \int \frac{{\left(N_{i1}\lambda_{\text{gi}}\right)}^{y_{\text{gi}1}}{\left(N_{i2}\lambda_{\text{gi}}\chi_{g}\right)}^{y_{\text{gi}2}}}{y_{\text{gi}1}!y_{\text{gi}2}!}e^{-N_{i1}\lambda_{\text{gi}}-N_{i2}\lambda_{\text{gi}}\chi_{g}}\; & \;\\ & \times f_{\lambda }(\lambda_{\text{gi}})d\lambda_{\text{gi}}\; & \;\\ = & \frac{\Gamma (y_{\text{gi}1}+y_{\text{gi}2}+\alpha_{g})}{y_{\text{gi}1}!y_{\text{gi}2}!\Gamma (\alpha_{g})}{\left(\frac{\beta_{g}}{\beta_{g}+N_{i1}+N_{i2}\chi_{g}}\right)}^{\alpha_{g}}\; & \;\\ & \times {\left(\frac{N_{i1}}{\beta_{g}+N_{i1}+N_{i2}\chi_{g}}\right)}^{y_{\text{gi}1}}{\left(\frac{N_{i2}\chi_{g}}{\beta_{g}+N_{i1}+N_{i2}\chi_{g}}\right)}^{y_{\text{gi}2}}\; & \; \end{array}$$

Therefore,

(5) $$\begin{array}{l} \;p(Y,\chi ,z|\theta )=\;\underset{g}{\prod }\left[\underset{i}{\prod }\left\{\frac{\Gamma (y_{\text{gi}1}+y_{\text{gi}2}+\alpha_{g})}{y_{\text{gi}1}!y_{\text{gi}2}!\Gamma (\alpha_{g})}\right)\right)\\ \;\times {\left(\frac{\beta_{g}}{\beta_{g}+N_{i1}+N_{i2}\chi_{g}}\right)}^{\alpha_{g}}\\ \;\times {\left(\frac{N_{i1}}{\beta_{g}+N_{i1}+N_{i2}\chi_{g}}\right)}^{y_{\text{gi}1}}\\ \left(\;\times {\left(\frac{N_{i2}\chi_{g}}{\beta_{g}+N_{i1}+N_{i2}\chi_{g}}\right)}^{y_{\text{gi}2}}\right\}\\ \left(\;\times p(\chi_{g}|z_{g},\theta )p(z_{g}|\theta )\right]. \end{array}$$

After integrating over the expected gene- and individual-specific relative baseline expression (*λgi*’s), the posterior density of unknown parameters is proportional to the product of likelihood and prior density.

p(χ,z,θ|Y)∝p(Y,χ,z|θ)p(θ)

We use the non-informative prior distributions for the unknown model parameters specified in the Methods Section.

### Parameter estimates by the Metropolis-Hastings algorithm (MCMC)

We infer the posterior distributions using the Gibbs sampling [43], which iteratively samples model paramters from the conditional distribution of each patermter given the other parameters. In this section, we describe the procedure for the posterior inference.

#### Step1

Update *α*_*g*_. The conditional distribution for *α*_*g*_ does not have a closed form expression. We use the Metropolis-Hastings algorithm to sample this parameter. More specifically, we update the parameter by proposing $\alpha_{g}^{\text{new}}\;∼\;N(\alpha_{g}^{\text{old}},\sigma_{\alpha }^{2})$ at each iteration, where *σ*_*α*_ is set to be 0.1. The proposal is accepted with probability *min*{1, *r*}, where *r* is the acceptance ratio.

$$r=\frac{p(z,\chi ,\alpha_{g}^{\text{new}},\theta_{-\alpha_{g}}^{\text{old}}|Y)}{p(z,\chi ,\alpha_{g}^{\text{old}},\theta_{-\alpha_{g}}^{\text{old}}|Y)}$$

where *θ* - *α*_*g*_*old* is the current values of the parameters except *α*_*g*_ and

$$\begin{array}{c} p(z,\chi ,\alpha_{g},\theta_{-\alpha_{g}}|Y)\;\propto \;\underset{i}{\prod }\frac{\Gamma (y_{\text{gi}1}+y_{\text{gi}2}+\alpha_{g})}{\Gamma (\alpha_{g})}\\ \;\times {\left(\frac{\beta_{g}}{\beta_{g}+N_{i1}+N_{i2}\chi_{g}}\right)}^{\alpha_{g}}. \end{array}$$

If the proposal is accepted, we replace the old *α*_*g*_ with the new one. Otherwise, *α*_*g*_ stays at the current value.

#### Step2

Update *β*_*g*_. Similar to sample *α*_*g*_, we propose *β*_*new*_ ∼ *N*(*β*_*old*_, *σβ*2), where *σ*_*β*_ is set to be 1. The acceptance ratio is calculated as

$$\;\begin{array}{c} p(z,\chi ,\beta_{g},\theta_{-\beta_{g}}|Y)\;\propto \;\underset{i}{\prod }\frac{\beta_{g}^{\alpha_{g}}}{{\left(\beta_{g}+N_{i1}+N_{i2}\chi_{g}\right)}^{y_{\text{gi}1}+y_{\text{gi}2}+\alpha_{g}}}. \end{array}$$

Similarly, *θ*_*-*_*β*_*g*_ is the vector of parameters except *β*_*g*_. For the evaluation of the acceptance probability, updated value of *α*_*g*_ in the Step 1 will be used.

#### Step3

Update (*χ*_*g*_, *z*_*g*_) by utilizing generalized Metropolis-Hastings. Lewin et al. [38] pointed out that *χ*_*g*_ and *z*_*g*_ have to be jointly estimated since the supporting space of *χ*_*g*_ depends on the choice of *z*_*g*_. For example, *χ*_*g*_ is a point mass at one if *z*_*g*_ = 0. To estimate a pair of (*χ*_*g*_, *z*_*g*_), they proposed the state *z*_*g*_ first and then updated *χ*_*g*_|*z*_*g*_. By utilizing this approach, we adopt the following steps to sample (*χ*_*g*_, *z*_*g*_).

(Step 3-1) Generate $z_{g}^{\text{new}}$ from the Bernoulli distribution, with $P(z_{g}^{\text{new}}=0)=\Pi_{0}^{\text{old}}$.

(Step 3-2) Then, $\chi_{g}^{\text{new}}$ is proposed from *LogNormal*(0, *V*_*g*_) if $z_{g}^{\text{new}}=0$. Otherwise, it is sampled from *LogNormal*(*M*_*g*_, *V*_*g*_). The mean and variance of the log-normal proposal distribution are computed from the observed counts. First, we collect individuals whose pre- and post-treatment counts are non-zero for each gene, separately. Then, *M*_*g*_ is computed as a median of $\log(\frac{y_{\text{gi}1}}{N_{i1}}/\frac{y_{\text{gi}2}}{N_{i2}}))$ for such individuals. The variance of these values can be used as *V*_*g*_, however, this estimate often gives an extreme value. In data analysis, we trim the estimates at 25th and 75th percentiles when the sample size is 10. For small sample case, the median of *V*_*g*_’s is used as the proposal variance.

Alternative description

Define $Q(\chi_{g}^{\text{new}},z_{g}^{\text{new}}|\chi_{g}^{\text{old}},z_{g}^{\text{old}})$ to be a proposal density from the current values $\left(\chi_{g}^{\text{old}},z_{g}^{\text{old}}\right)$ to the proposed values. In our approach, the proposal density does not depend on the current values, *i.e.*, we use the independence chain Metropolis-Hastings. The proposal distribution is given by

$$\begin{array}{lll} \;Q\left(\chi_{g}^{\text{new}},z_{g}^{\text{new}}|\chi_{g}^{\text{old}},z_{g}^{\text{old}}\right)∼ & \Pi_{0}^{\text{old}}\text{LN}(0,V_{g})I\left(z_{g}^{\text{new}}=0\right)\; & \;\\ & +\Pi_{1}^{\text{old}}\text{LN}(M_{g},V_{g})I\left(z_{g}^{\text{new}}\;=\;1\right)\; & \; \end{array}$$

The acceptance probabilty is *min*{1, *r*} where *r* is one of followings:

$$\begin{array}{ccccc} z_{g}^{\text{old}}= & 1,z_{g}^{\text{new}}=1 & : & r=\frac{LN_{1}(\chi_{g}^{\text{new}})t(\chi_{g}^{\text{new}})}{LN_{1}(\chi_{g}^{\text{old}})t(\chi_{g}^{\text{old}})}\; & \;\\ & & & \;\;\times \frac{\text{LN}(\chi_{g}^{\text{old}};M_{g},V_{g})}{\text{LN}(\chi_{g}^{\text{new}};M_{g},V_{g})}\; & \;\\ z_{g}^{\text{old}}= & 1,z_{g}^{\text{new}}=0 & : & r=\frac{LN_{0}(\chi_{g}^{\text{new}})t(\chi_{g}^{\text{new}})}{LN_{1}(\chi_{g}^{\text{old}})t(\chi_{g}^{\text{old}})}\; & \;\\ & & & \;\;\times \frac{\text{LN}(\chi_{g}^{\text{old}};M_{g},V_{g})}{\text{LN}(\chi_{g}^{\text{new}};0,V_{g})}\; & \;\\ z_{g}^{\text{old}}= & 0,z_{g}^{\text{new}}=1 & : & r=\frac{LN_{1}(\chi_{g}^{\text{new}})t(\chi_{g}^{\text{new}})}{LN_{0}(\chi_{g}^{\text{old}})t(\chi_{g}^{\text{old}})}\; & \;\\ & & & \;\;\times \frac{\text{LN}(\chi_{g}^{\text{old}};0,V_{g})}{\text{LN}(\chi_{g}^{\text{new}};M_{g},V_{g})}\; & \;\\ z_{g}^{\text{old}}= & 0,z_{g}^{\text{new}}=0 & : & r=\frac{LN_{0}(\chi_{g}^{\text{new}})t(\chi_{g}^{\text{new}})}{LN_{0}(\chi_{g}^{\text{old}})t(\chi_{g}^{\text{old}})}\; & \;\\ & & & \;\;\times \frac{\text{LN}(\chi_{g}^{\text{old}};0,V_{g})}{\text{LN}(\chi_{g}^{\text{new}};0,V_{g})}\; & \; \end{array}$$

where $t(\chi_{g})=\underset{i}{\prod }\frac{\chi_{g}^{y_{\text{gi}2}}}{{\left(\beta_{g}+N_{i1}+\chi_{g}N_{i2}\right)}^{y_{\text{gi}1}+y_{\text{gi}2}+\alpha_{g}}}$, *LN*_0_ is a probability density function for log-normal distribution with mean zero and variance $\sigma_{0}^{2,\text{old}}$. Similarly, *LN*_1_ is a log-normal density centered at $\mu_{1}^{\text{new}}$ and variance $\sigma_{1}^{2,\text{old}}$.

#### Step4

Update $\sigma_{0}^{2}$, *μ*_1_, $\sigma_{1}^{2}$, which are hyper-paramaters from the distribution of *χ*_*g*_. Since it has a closed form for the posterior density conditional on all other parameters, we can directly sample those parameters.

$$\begin{array}{l} \sigma_{0}^{2,\text{new}}∼\text{InvGamma}\left(\frac{♯(z_{g}=0)}{2},\;\;\frac{1}{2}\underset{z_{g}=0}{\sum }\log{\left(\chi_{g}\right)}^{2}\right)\\ \mu_{1}^{\text{new}}∼\text{Normal}\left(\frac{\underset{z_{g}=1}{\sum }\log{\left(\chi_{g}\right)}^{2}}{♯(z_{g}=1)},\;\;\frac{\sigma_{1}^{2}}{♯(z_{g}=1)}\right)\\ \sigma_{1}^{2,\text{new}}∼\text{InvGamma}\left(\frac{♯(z_{g}=1)}{2},\;\;\frac{1}{2}\underset{z_{g}=1}{\sum }{\left(\log\chi_{g}-\mu_{1}\right)}^{2}\right) \end{array}$$

where $♯(z_{g}=0)=\underset{g}{\sum }I(z_{g}=0)$ and $♯(z_{g}=1)=\underset{g}{\sum }I(z_{g}=1)$.

#### Step5

Update the mixing proportions (*Π*_0_, *Π*_1_). We assume a Dirichilet prior for the mixture probabilities. Using Gibbs sampling scheme, these weight parameters are updated from *Dir*(1 + *♯*(*z*_*g*_ = 0), 1 + *♯*(*z*_*g*_ = s1)).

## Competing interests

The authors declare that they have no competing interests.

## Authors’ contributions

LMC developed and implemented the proposed model, performed statistical analysis, and drafted the manuscript. JPF participated in model development and helped manuscript preparation. WZ processed the WNV data and participated in data analysis. FQ, VB, and RRM performed WNV experiment. HZ designed and coordinated the study and helped draft the manuscript. All authors read and approved the final manuscript.
